# Endothelin-1 Promotes Survival and Chemoresistance in Chronic Lymphocytic Leukemia B Cells through ET_A_ Receptor

**DOI:** 10.1371/journal.pone.0098818

**Published:** 2014-06-05

**Authors:** Rossana Maffei, Jenny Bulgarelli, Stefania Fiorcari, Silvia Martinelli, Ilaria Castelli, Vanessa Valenti, Davide Rossi, Goretta Bonacorsi, Patrizia Zucchini, Leonardo Potenza, Daniele Vallisa, Valter Gattei, Giovanni Del Poeta, Francesco Forconi, Gianluca Gaidano, Franco Narni, Mario Luppi, Roberto Marasca

**Affiliations:** 1 Hematology Division, Department of Medical and Surgical Sciences, University of Modena and Reggio Emilia, Modena, Italy; 2 Hematology Division, Piacenza Hospital, Piacenza, Italy; 3 Hematology Division, Department of Clinical and Experimental Medicine, Amedeo Avogadro University of Eastern Piedmont, Novara, Italy; 4 Clinical and Experimental Onco-Hematology Unit, Centro di Riferimento Oncologico, I.R.C.C.S., Aviano (PN), Italy; 5 Hematology Division, S.Eugenio Hospital and University of Tor Vergata, Rome, Italy; 6 Cancer Sciences Unit, CRUK Clinical Centre, University of Southampton, Southampton, United Kingdom; 7 Hematology Division, Department of Clinical Medicine and Immunological Sciences, University of Siena, Siena, Italy; Wayne State University, United States of America

## Abstract

The endothelin axis, comprising endothelins (ET-1, ET-2 and ET-3) and their receptors (ET_A_R and ET_B_R), has emerged as relevant player in tumor growth and metastasis. Here, we investigated the involvement of ET-1/ET_A_R axis in chronic lymphocytic leukemia (CLL). CLL cells expressed higher levels of ET-1 and ET_A_ receptor as compared to normal B cells. ET-1 peptide stimulated phosphoinositide-3-kinase and mitogen-activated protein kinase signaling pathways, improved survival and promoted proliferation of leukemic cells throughout ET_A_R triggering. Moreover, the blockade of ET_A_R by the selective antagonist BQ-123 inhibited the survival advantage acquired by CLL cells in contact with endothelial layers. We also found that blocking ET_A_R via BQ-123 interferes with ERK phosphorylation and CLL pro-survival effect mediated by B-cell receptor (BCR) activation. The pro-apoptotic effect of phosphoinositide-3-kinase δ inhibitor idelalisib and mitogen-activated protein kinase inhibitor PD98059 was decreased by the addition of ET-1 peptide. Then, ET-1 also reduced the cytotoxic effect of fludarabine on CLL cells cultured alone or co-cultured on endothelial layers. ET_A_R blockade by BQ-123 inhibited the ET-1-mediated protection against drug-induced apoptosis. Lastly, higher plasma levels of big ET-1 were detected in patients (n = 151) with unfavourable prognostic factors and shorter time to first treatment. In conclusion, our data describe for the first time a role of ET-1/ET_A_R signaling in CLL pathobiology. ET-1 mediates survival, drug-resistance, and growth signals in CLL cells that can be blocked by ET_A_R inhibition.

## Introduction

Chronic lymphocytic leukemia (CLL) is the most common leukemia in adults in the Western countries. CLL is caused by the accumulation of a long-lived antigen-experienced B cell clone, of which a small fraction is represented by actively proliferating cells with approximately 1-2% of cells newly generated each day [1]. The small proportion of proliferating CLL cells is thought to replenish leukemic population inside specific structures known as proliferation centers, which are localized in lymph nodes and bone marrow. Bidirectional interactions with surrounding non-transformed cells of stromal and immune compartments inside proliferation centers prolong CLL survival, mediate proliferation stimuli, and protect cells from the effect of chemotherapeutics [2]. In addition, CLL activation inside tissue microenvironments may induce genetic instability and contribute to progression towards a more malignant phenotype through the acquisition of additional genetic lesions [3].

The most promising novel therapeutic approaches emerging in CLL clinical trials have been developed to target CLL microenvironment, by interfering with homing and migration of CLL cells [4]. Indeed, recirculation of leukemic cells from peripheral blood to protective niches has emerged as a relevant feature in the progression of the disease, with the involvement of several molecules such as chemokines, their receptors, adhesion molecules and enzymes able to digest the extracellular matrix. Inside tissues, CLL cells also experience a chronic antigen contact that implies the engagement of the B cell receptor (BCR) signaling, leading to activation of downstream pro-survival signaling molecules such as nuclear factor-kB, Raf, mitogen-activated protein kinase MEK and extracellular signal regulated kinase (ERK) [4]. Moreover, increasing evidence suggests that angiogenesis can play a role in CLL patho-physiology [5]. CLL-infiltrated tissues are characterized by high vascularization levels with abnormal microvessels mainly localized near proliferating CLL subclone [6]. Patients with adverse clinical outcome show more vascularized CLL-infiltrated tissues and increased angiogenesis-related factors in plasma [7]. Furthermore, CLL contact with endothelial cells mediates survival, proliferation and drug-resistance [6], [8]–[10]. Among the most up-regulated genes activated in CLL cells after contact with endothelial cells, we recently reported Endothelin-1 (ET-1) with a 9-fold increase [8].

ET-1 is a 21-aa peptide that mediates its action by activating two G-protein-coupled receptor (GPCR) subtypes, ET_A_ and ET_B_ receptors [11]. Major pathways and effectors downstream of ET receptors include mitogen activated protein kinases (MAPKs) and phosphatidylinositol 3- kinase (PI3K)/AKT signaling pathways, adenylyl cyclase and phospholipases (PLCβ and PLA2). Synthesis of the biologically active ET-1 peptide is a multistep process. The primary translation product of *EDN1* gene is the 212-aa preproET-1, which is cleaved by an endothelin converting enzyme (ECE-1) to form the 38-aa big ET-1 and then to the biologically active 21-aa ET-1 peptide [11]. In addition to its role as a potent endogenous vasoconstrictor and mediator of cardiovascular and renal disorders, the endothelin axis has emerged as relevant player in tumor growth and metastasis by regulating cell survival, angiogenesis, tumor-infiltrating immune cells, epithelial-to-mesenchymal transition, invasion and metastatic dissemination [12]. Endothelin receptor blockade represents the most promising approach in controlling the pleiotropic activities of ET-1 [13].

We evaluated whether ET-1 signaling pathway may be involved in CLL pathobiology. Our findings demonstrate a role of ET-1 signaling via ET_A_R in CLL prolonged survival, proliferation and drug-resistance. The effects are mediated by the activation of PI3K/AKT and ERK/MAPK signaling pathways. Interestingly, the blockade of ET_A_R via BQ-123 interferes with the pro-survival signal and ERK phosphorylation induced by BCR triggering. We also demonstrated that ET-1 signaling attenuates the effect of idelalisib, an inhibitor of PI3Kδ and PD98059, a MEK inhibitor. Moreover, ET-1/ET_A_R axis plays a role in CLL interaction with endothelial cells, suggesting that ET-1 may contribute to establish a nursing and protective niche in infiltrated tissues. A range of specific and selective ET_A_ antagonists have undergone preclinical and clinical studies showing promising results in some cancer settings mainly in combination with cytotoxic drugs. Collectively, our findings suggest that ET-1/ET_A_R axis may represent a novel therapeutic target in CLL.

## Materials and Methods

### Ethics statement

Written informed consent was obtained in accordance with the Declaration of Helsinki with a protocol approved by the local Institutional Review Board (Comitato Etico Provinciale di Modena, protocol#1298-39/10).

### CLL patients and samples

Blood samples were collected at diagnosis from 151 CLL patients fulfilling standard clinical, morphological and immunophenotypic criteria [14] at the Divisions of Hematology of Novara (n = 73), Modena (n = 42), Siena (n = 26), and Rome (n = 10). Plasma samples were obtained by blood centrifugation at 2000 rpm for 15 minutes, then centralized to the Hematology Unit of Modena for big ET-1 quantification. Plasma samples from the same patient at different time points during follow-up were also evaluated in 8 CLL cases. Peripheral blood mononuclear cells (PBMCs) collected from untreated CLL patients were isolated by density gradient centrifugation (Ficoll, Pharmacia LKB Biotechnology, Piscataway, NY, USA) and cryopreserved in RPMI-1640 medium, 50% fetal bovine serum (FBS), and 10% DMSO and stored in liquid nitrogen until use. Normal B lymphocytes were also obtained from buffy coats of healthy donors (HD) from the blood bank of Modena Hospital. To enrich for CLL and normal B cells, PBMCs were incubated with CD19Microbeads (Miltenyi Biotec, Auburn, CA, USA), obtaining a purity >99% as assessed by flow cytometry using PE-conjugated CD19 Ab (Miltenyi Biotec). All experiments were performed on highly purified CLL and normal B cell samples.

### Cell culture conditions

Purified CLL cells were suspended at a final concentration of 1×10^6^/ml in RPMI medium with 10% FBS and then plated in 24-well plates. Human Umbilical Vein Endothelial Cells (HUVEC, Cascade Biologics, Life Technologies, Carlsbad, CA, USA) were cultured as previously described [8]. For co-culture experiments, HUVEC cells were incubated until reaching 70% confluence and CLL cells were then seeded onto HUVEC layer. Leukemic cells were also stimulated with recombinant ET-1 peptide (Calbiochem, Merck, Darmstadt, Germany) at 100 nM. To evaluate drug-resistance, cells were exposed to fludarabine (2-Fluoroadenine 9-B-D-arabinofuranoside, Sigma-Aldrich, St. Luis, MO, USA) at dose of 1 µM, idelalisib (GS-1101) (Selleckchem, Houston, TX, USA) at 0.5 µM, and PD98059 (Sigma-Aldrich) at 50 µM. When indicated, CLL cells were pre-incubated with ET_A_R antagonist BQ-123 (Sigma-Aldrich) for 20 minutes at 37°C (0.1 µM or 1 µM) before EC co-culture or treatments with fludarabine or ET-1. Recombinant human interleukin 2 (IL-2) (100 IU/mL) and CpG oligonucleotides (1 µg/mL; ODN2006, InvivoGen, S.Diego, CA, USA) were also used. To determine the effect of BQ-123 on CLL survival mediated by BCR signaling, CLL cells (3×10^6^/mL) were previously incubated at 37°C with or without 0.1 µM BQ-123 for 20 minutes, then stimulated with 10 µg/mL of anti-IgM (Sigma-Aldrich) in complete RPMI medium. At the indicated time points, CLL cells were collected by removal of the supernatant and then being assayed. In co-culture experiments, the number of HUVEC cells contaminating the supernatants derived from the spontaneous detachment of apoptotic cells and increased during co-culture time, reaching 0.6–0.9% after 7 days, as assessed by flow cytometric stainings with APC-conjugated CD146 Ab (Miltenyi Biotech) and Annexin V (eBioscience, San Diego, CA, USA). The effect of HUVEC contamination in the analyzed samples was excluded by different strategies, as indicated. Conditioned media (CM) were also collected by centrifugation at 1600 rpm for 10 minutes and stored at −20°C before being assayed.

### Flow cytometry

Cells were stained with rabbit polyclonal ET_A_R antibody (Abgent, S.Diego, CA, USA) for 30 min in ice followed by FITC-conjugated Goat anti-rabbit Ig for 30 min in ice (Becton Dickinson, San José, CA, USA) and APC-conjugated CD19 (Miltenyi Biotec) for 10 min at room temperature. Apoptotic cell death of CLL cells was analyzed using Annexin V-FITC and Propidium Iodide (PI) staining (eBioscience). HUVEC cells were excluded by a lymphocyte gate set according to the different relative size and granularity (forward scatter and side scatter). Viability was defined as the percentage of Annexin V-/PI- cells (lower left quadrant). Events were acquired using a FACSCalibur cytometer (Becton Dickinson) and then analyzed by FlowJo Software (Tree Star, Ashland, OR). In addition, CFSE (5-[and 6]-Carboxyfluorescein diacetate succinimidyl ester; eBioscience) dilution assay was used to trace cell division by flow cytometry. CD19+ CLL cells, stained with CFSE, were incubated or not with BQ-123 (0.1 µM) and then plated onto the HUVEC layer. The proliferative measure was evaluated after 4 days, gating the CD19+ alive CLL cells and analyzed using FlowJo software. CLL cell proliferation was also evaluated by cell cycle analysis. CD19+ CLL cells were treated or not with ET-1 (100 nM) overnight and then incubated in citrate buffer containing 10 mg/ml PI (Sigma) and 100 µg/ml RNase. Cell cycle profiles were analyzed using Modfit LT software (Verity Software House, Topshem, ME, USA). To exclude debris and aggregates, we gated live cells and selected single cells according to FL2-A vs FL2-W plot. CLL cells stimulated with rh-IL-2 and CpG oligonucleotides were used as positive control in CFSE dilution assay and cell cycle analysis.

### MTT assays

CLL activation was monitored using a yellow tetrazolium MTT assay (Trevigen, Gaithersburg, MD, USA). In this assay, dehydrogenases expressed by metabolically active cells convert MTT (3-[4,5-dimethylthiazol-2-yl]-2,5-dyphenyl-tetrazolium bromide) into intracellular purple formazan. CLL cells (100 µL/well) were collected from cultures and then allowed to adhere into a 96-well plate by centrifugation at 1000 rpm for 10 min. Cells were then incubated with MTT at 37°C for 24 hours, followed by a 4 h-incubation with 100 µL detergent reagent. Absorbance readings were performed at 570 nm in a microplate reader (Infinite M200, Tecan, Männedorf, Switzerland).

### Enzyme-linked immunosorbent assays

Big ET-1 levels in conditioned media and plasma samples were measured using Big Endothelin-1 (human) EIA kit (Enzo Life Sciences, Farmingdale, NY, USA). The mean minimum detectable dose was 0.23 pg/mL. Each sample was tested in duplicate and concentrations were reported in pg/mL.

### Real-time PCR

RNA was extracted with the RNeasy Plus Mini kit (Qiagen, Valencia, CA, USA). Then, RNA (100 ng) was reverse transcribed using Transcription High fidelity cDNA Synthesis kit (Roche Applied Science, Penzberg, Germany). ET-1 and ET_A_R mRNA were amplified by using LightCycler 480 SYBR Green I Master (Roche). Primers were as follows: ET-1, forward 5′-TCTCTGCTGTTTGTGGCTTG-3′, reverse 5′-GAGCTCAGCGCCTAAGACTG-3′; ET_A_R, forward 5′-TGGTGTGCACTGCGATCTTC-3′, reverse 5′-GCAATTCTCAAGCTGCCATTC-3′; GAPDH, forward 5′-GAAGGTGAAGGTCGGAGTC-3′, reverse 5′-GAAGATGGTGATGGGATTTC-3′. All samples were analyzed in real time on LightCycler 480 v.2 (Roche) in duplicate, as previously described [15].

### Western blot

After 1 h starvation at 37°C in RPMI medium with 2% FBS, freshly isolated CLL cells were cultured in suspension and stimulated with 100 nM ET-1 peptide (Calbiochem, Merck) for 4 hours or 10 µg/mL anti-IgM for 10 minutes. When indicated, CLL cells were pre-incubated with ET_A_R antagonist BQ-123 (Sigma) for 20 minutes at 37°C before stimulation. Cells were lysed on ice for 10 minutes with lysis buffer supplemented with dithiothreitol and protease inhibitor cocktail (BioVision, Milpitas, CA, USA). Proteins (100 µg/lane) were electrophoresed on 4-20% SDS-polyacrylamide gradient gels and transferred to nitrocellulose membranes (Bio-Rad Laboratories, Hercules, CA, USA). Membranes were immunoblotted with the following primary antibodies: anti-ET_A_R rabbit Ab (1∶1000; Abcam, Cambridge, MA, USA) anti-Akt rabbit mAb (1∶1000; Cell Signaling Tech, Beverly, MA, USA) or anti-phospho-Akt rabbit mAb (Ser 473, 1∶1000; Cell Signaling Tech); anti-p44/42 MAPK (Erk1/2) rabbit mAb (1∶1000; Cell Signaling Tech) or anti-phospho-p44/42 MAPK (Erk1/2) rabbit mAb (Thr202/Tyr204, 1∶2000; Cell Signaling Tech); anti-βactin mouse mAb (ab6276, 1∶10000; Abcam). Then, membranes were incubated with species-specific horseradish peroxidase (HRP)-conjugated secondary antibody (1∶50000; GE Healthcare, Uppsala, Sweden). Blots were developed using SuperSignal West Pico Chemiluminescent Substrate (Thermo Scientific, Rockford, IL, USA). Images were acquired by Chemidoc XRS+ and analyzed using Image Lab Software v.3.0 (Bio-Rad Laboratories).

### Immunohistochemistry

Sections from lymph nodal biopsies and prostate cancer biopsies were incubated with mouse monoclonal Anti-Endothelin1 antibody (Calbiochem, Merck, Darmstadt, Germany, dilution 1∶250) or with rabbit polyclonal ET_A_R antibody (Abgent, dilution 1∶50). Detection of bound antibody was performed with alkaline phosphatase method using a streptavidin-biotin alkaline phosphatase complex kit (REAL Detection System, Dako, Glostrup, Denmark). The alkaline phosphatase reaction was then developed with Permanent Red (Dako) as chromogen.

### Statistical analyses

Data were analyzed using SPSS version 20.0 (SPSS, Chicago, IL, USA). The cut-off point for big ET-1 levels was selected according to receiver operating characteristic (ROC) analysis using treatment as state variable, and the Youden's index was calculated utilizing the sensitivity and specificity derived from ROC analysis. Time to first treatment (TTFT) was estimated using product-limit (Kaplan-Meier) method and the curves were compared between groups using log-rank test. The 2-tailed unpaired or paired Student *t* test (*p<0.05, **p<0.01, ***p<0.001) was used to compare data between 2 experimental groups. Standard error of the mean (SEM) is depicted as error bars.

## Results

### CLL cells express ET-1 and ET_A_ receptor

**Figure 1:**
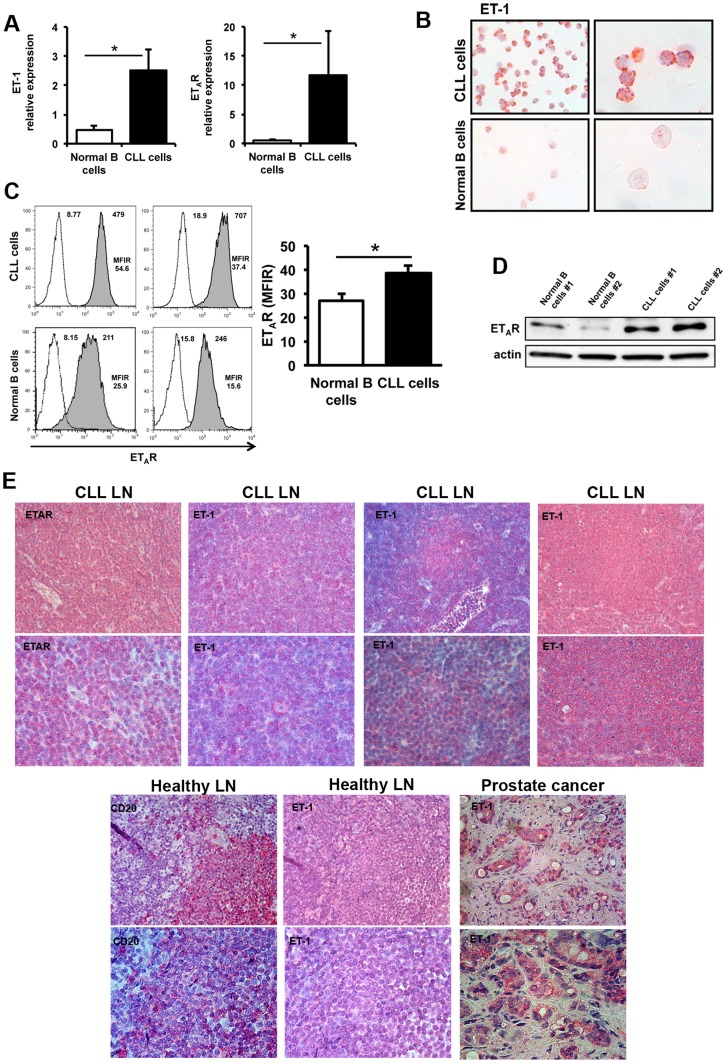
CLL cells express ET-1 and ET_A_ receptor. (A) ET-1 and ET_A_R expression levels were evaluated by quantitative reverse-transcription PCR on CLL cells (n = 10) and normal B lymphocytes (n = 6) purified from peripheral blood. Histograms depict mean±SEM of ET-1 and ET_A_R relative expression. Results were normalized to the expression of GAPDH housekeeping gene. CLL cells show higher expression levels of both ET-1 and ET_A_R mRNA compared to normal B cells (*p<0.05). (B) CLL cells or normal B lymphocytes purified from peripheral blood were allowed to adhere and then stained with anti-ET-1 antibody. A representative case of 3 independent CLL samples and 3 normal B cell samples is shown. Original magnification, 400X for left panels, and 1000X for right panels. CLL cells show more intense stainings of ET-1 peptide than normal B cells. (C) Displayed are flow cytometric histograms depicting the relative fluorescence intensity of 2 CLL samples and 2 normal B-cell samples stained with anti-CD19 and anti-ET_A_R Abs. Mean fluorescence intensity ratio (MFIR) is displayed above the histograms and is calculated by dividing the mean fluorescence intensity for ET_A_R by the mean fluorescence of the isotype control. Histograms on the right summarize MFIR data of B cells from 7 CLL patients and 6 normal controls. Data are presented as mean±SEM. Increased expression of ET_A_R on the surface of CLL cells was measured as compared to normal B cells (*p<0.05). (D) The immunoblots depict higher ET_A_R expression levels in CLL cells than in normal B lymphocytes purified from peripheral blood detected by Western blot analysis. (E) Immunohistochemical evaluation of CLL-infiltrated lymph nodes (CLL LN) (n = 4) stained with antibodies against ET-1 or ET_A_ receptor showing positive CLL cells. A representative staining of lymph nodes from healthy donors (n = 3) is displayed, showing a faint ET-1 expression on normal B lymphocytes identified by CD20 staining. Prostate cancer is shown as positive control. Original magnification, 200X in the above panels and 400X in the bottom panels.

CLL cells circulating in peripheral blood expressed ET-1 and exposed ET_A_R on cell surface (Figure 1). Comparing the expression levels between purified CLL cells (n = 10) and normal B cells (n = 6) collected from peripheral blood, we found that ET-1 and ET_A_R were significantly over-expressed in CLL cells (Figure 1A). Moreover, we stained CLL cells (n = 3) and normal B cells (n = 3) with anti-ET-1 antibody, revealing an increased amount of ET-1 peptide in CLL as compared to normal B lymphocytes (Figure 1B). Then, we cultured purified CLL cells (n = 13) and normal B cells (n = 4) for 72 hours and we quantified the big ET-1 protein, 38-aa precursor of ET-1, in conditioned media by ELISA. CLL secreted a mean level of ET-1 precursor of 4.0±1.1 pg/mL, ranging from 0.9 to 12.8 pg/mL. Conversely, we did not detect ET-1 secretion by normal B cells (data not shown). Interestingly, we observed that big ET-1 was mostly secreted by leukemic cells derived from patients with unfavorable unmutated IGHV genes (mean±SEM, 5.2±1.5 pg/mL) compared to mutated ones (1.2±0.14 pg/mL) (p = 0.027) (Figure S1 in File S1).

Furthermore, we quantified ET_A_R on the surface of CLL (n = 7) and normal B lymphocytes (n = 6). CLL cells expressed ET_A_R at higher levels (mean fluorescence intensity ratio, MFIR = 39±3) as compared to normal B cells (MFIR = 27±3) (p = 0.017, Figure 1C). No differential expression of ET_A_R was detected between mutated and unmutated IGHV CLL subsets. In addition, CLL cells were positive for ET-1 and ET_A_R when infiltrating lymph nodes. ET-1 staining was more intense in CLL cells localized inside proliferation centers. Prostate cancer was used as positive control (Figure 1E).

### Blocking ET_A_R reduces CLL survival and interferes with B cell receptor (BCR) signaling

**Figure 2:**
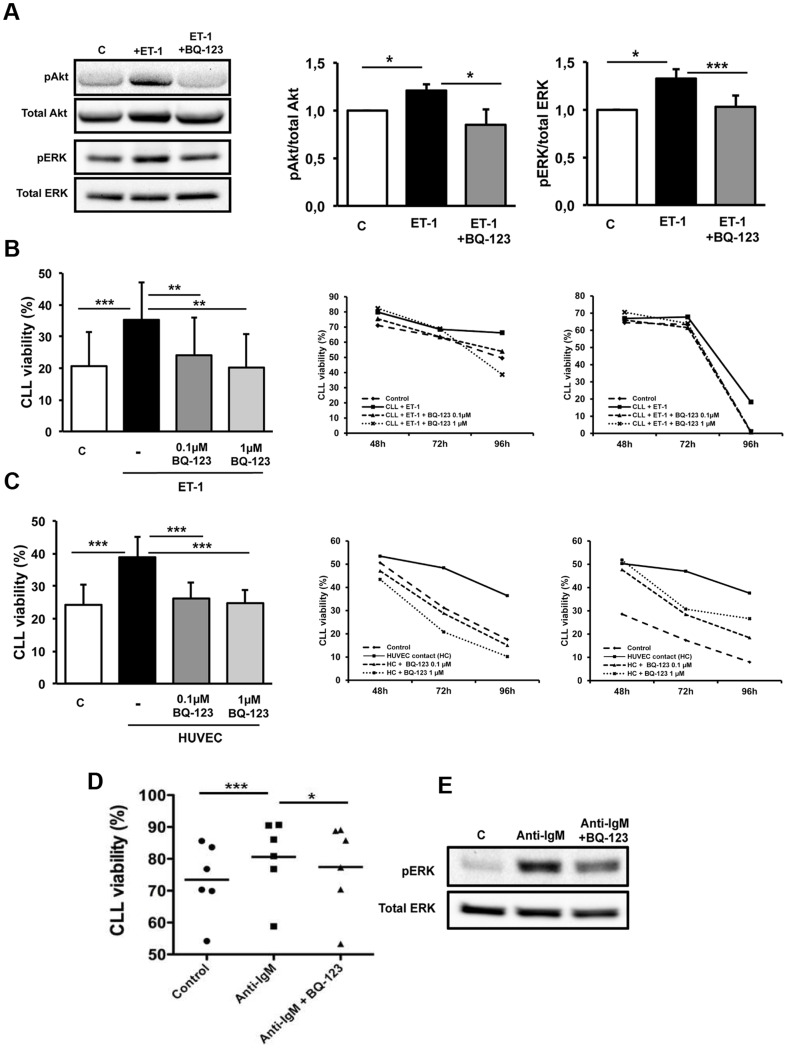
ET-1 mediates survival signal on CLL cells. (A) Serum-starved CLL cells were stimulated with 100 nM ET-1 for 1 hour, pretreating or not cells with 0.1 µM BQ-123 (20 min). Western blot analysis of CLL cells was performed with anti-phospho-Akt, anti-Akt, anti-phospho-ERK, anti-ERK and anti-β-actin antibodies. ET-1 stimulates Akt and ERK phosphorylation through ET_A_R. The immunoblots depict Akt and ERK activation in a representative case. Histograms represent densitometric quantification (pAkt/total Akt ratio and pERK/total ERK ratio) of bands relative to phospho-Akt, total Akt, phospho-ERK and total ERK normalized on β-actin. Data are presented as mean±SEM of 6 CLL patients relative to unstimulated control (*p<0.05, ***p<0.001). (B) CLL cells (n = 6) were cultured with the addition of recombinant ET-1 peptide at 100 nM for 96 hours. CLL cell viability was assessed by flow cytometry using Annexin V and PI staining. A lymphocyte gate was set according to the different relative size and granularity (forward scatter and side scatter) and viable cells were defined as Annexin V-/PI-. Histograms represent mean±SEM at 96 h of 6 CLL patients evaluated in 3 independent experiments. Two representative cases are depicted on the right, showing cell viability at 48 h, 72 h and 96 h. Control is defined as viability of CLL cells cultured alone in complete medium. ET-1 stimulation improves CLL survival. The effect is inhibited by pre-treating CLL cells with BQ-123 (0.1–1 µM) (**p<0.01, ***p<0.001). (C) CLL cells (n = 11), pretreated or not with BQ-123 (0.1–1 µM), were cultured on HUVEC cell layer for 96 hours. CLL cell viability was assessed by flow cytometry, as described for panel B. Histograms represent data as mean±SEM of 11 CLL patients evaluated in 4 independent experiments. Two representative cases are depicted on the right, showing cell viability at 48 h, 72 h and 96 h. Control is defined as viability of CLL cells cultured alone in complete medium. Note that BQ-123 significantly reduces the CLL pro-survival effect mediated by HUVEC contact (***p<0.001). (D) CLL cells were stimulated with 10 µg/mL of anti-IgM for 48 hours with or without 0.1 µM BQ-123. Dot plots represent cell viability of 6 CLL patients evaluated in 3 independent experiments. The blockade of ET_A_R reduces the pro-survival effect of BCR triggering in CLL cells (*p<0.05). (E) Serum-starved CLL cells were pretreated or not with 0.1 µM BQ-123 for 20 minutes before stimulation with anti-IgM for 10 minutes. The immunoblots depict ERK phosphorylation in a representative case.

Serum-starved CLL cells (n = 6) were treated with 100 nM ET-1 for 1 hour. Exposure to ET-1 was also performed after ET_A_R blocking by pretreating cells with 0.1 µM BQ-123 for 20 minutes. When CLL cells were stimulated with ET-1 peptide, we detected the activation of PI3K/Akt and ERK/MAPK signaling pathways through ET_A_ receptor triggering (Figure 2A). To explore the role of ET-1 in preserving CLL survival, leukemic cells (n = 6) were cultured alone for 4 days in complete medium in presence of ET-1 peptide at 100 nM. CLL cell viability increased upon ET-1 stimulation, from 21%±11% in untreated CLL to 35%±12% in ET-1 treated CLL cells (p = 0.0008, Figure 2B). Treatment with BQ-123 at doses of 0.1 µM or 1 µM did not exert any cytotoxic effect on CLL cells (n = 3) cultured alone from 24 to 96 hours (data not shown). However, if stimulated with ET-1 peptide, the blockade of ET_A_R by 0.1 µM and 1 µM BQ-123 abrogated the ET-1-induced apoptosis resistance, decreasing CLL viability to 24%±12% and 20%±11% respectively (p = 0.003 and p = 0.004, Figure 2B and Figure S2 in File S1).

We previously demonstrated that CLL cells acquire a survival advantage when cultured in direct contact with endothelial cells [8]. Conditioned media collected after 72 h co-culture (n = 13) were enriched of big ET-1 peptides secreted by both endothelial and leukemic cells (mean±SEM, 487.6±24.5 pg/mL). In addition, when co-cultured on endothelial layer, the expression of ET_A_R was maintained at high levels by CLL cells (data not shown), indicating that ET-1 signaling may be relevant in EC/CLL interaction. Thus, we argued whether ET-1/ET_A_R axis could be involved in survival advantage acquired by CLL when cultured together with endothelial cells. CLL cells (n = 11), pretreated or not with BQ-123 for 20 minutes, were cultured in direct contact with endothelial layer (HC condition) for 96 hours. We confirmed that CLL cells acquire a survival advantage when co-cultured with endothelial cells, increasing the percentage of viable cells from 24%±6% in CLL alone to 39%±6% in co-culture (p = 0.0005). Blocking ET_A_R on CLL, by pretreating cells with 0.1 µM and 1 µM BQ-123 before co-culture, abrogated the pro-survival effect of endothelial cell contact, decreasing CLL viability to 26%±5% and 25%±4% respectively (p = 0.0005 and p = 0.0008, Figure 2C and Figure S2 in File S1). Interestingly, CLL cells with mutated or unmutated IGHV genes showed similar responsiveness to ET_A_R inhibitor treatment (Figure S3 in File S1). Inhibition of ET_A_R induced apoptosis in CLL harboring 17p deletion (n = 2), from 56% and 67% of viable cells in contact with endothelial layer to 38% and 36% in the presence of BQ-123.

Activation of CLL cells via BCR sets in motion a cascade of intracellular signaling events, including PI3K and MAPK pathways, that results in enhanced CLL survival. To determine the effects of BQ-123 on CLL viability mediated via BCR, we stimulated cells (n = 6) with anti-IgM in presence of 0.1 µM BQ-123. As shown in Figure 2D, anti-IgM stimulation increased CLL viability from 73%±5% to 81%±5% (p = 0.0006). Moreover, the blockade of ET_A_R signaling by BQ-123 decreased the pro-survival effect of BCR triggering to 77±6% (p = 0.02). In addition, we found that BQ-123 reduced ERK phosphorylation in response to anti-IgM stimulation (Figure 2E).

### ET-1 signaling mediates chemo-resistance through ET_A_R

**Figure 3:**
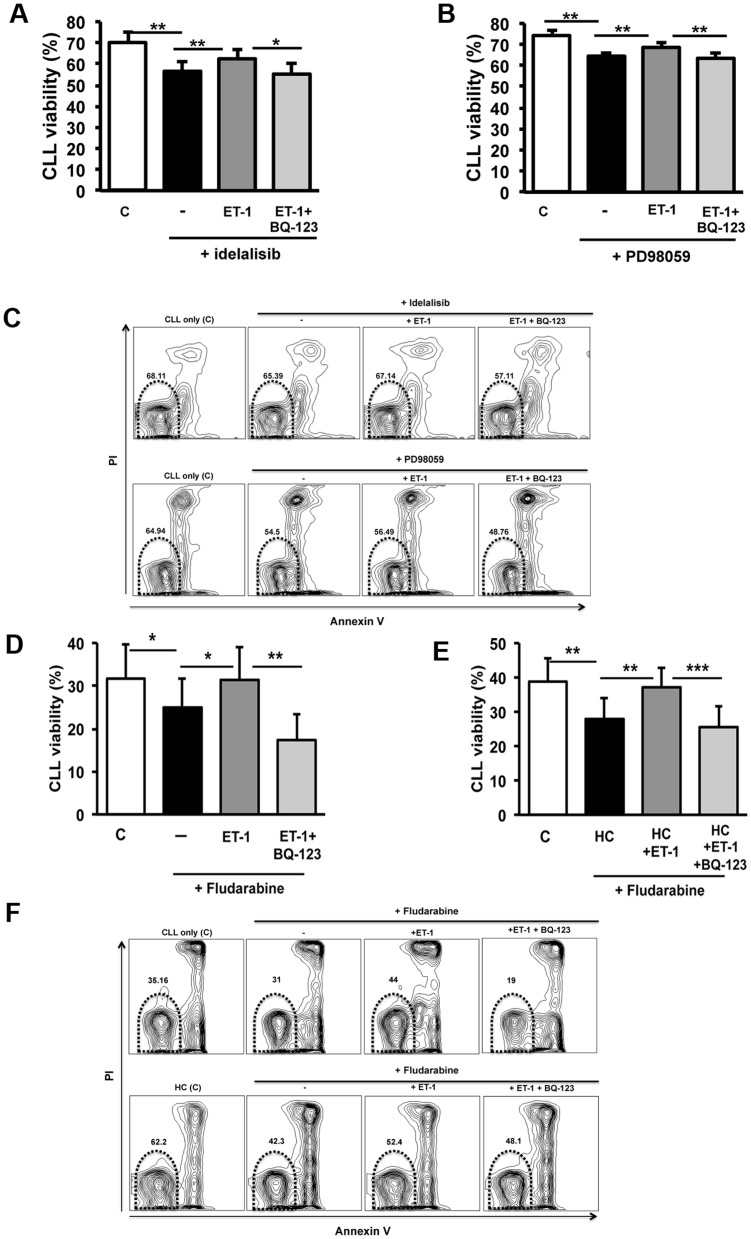
ET-1 inhibits the pro-apoptotic effect of idelalisib, MEK inhibitor and fludarabine. CLL cells (n = 8) were treated with idelalisib at 0.5 µM (panel A) or PD98059, a MEK inhibitor, at 50 µM (panel B). When indicated, CLL cells were also incubated with BQ-123 at 0.1 µM before ET-1 stimulation. CLL cell viability was assessed by flow cytometry by using Annexin V and PI staining. CLL viable cells were defined as Annexin V-/PI-. Histograms represent cell viability as mean±SEM at 48 hours of 8 CLL patients evaluated in 4 independent experiments. Control is defined as CLL cells cultured without any treatment. Flow cytometric contour plots of one representative case are shown in panel C. The gates in the plots exemplify viable CLL cells (Annexin V-/PI-, circled). ET-1 signaling reduces CLL sensitivity to both idelalisib and PD98059 (**p<0.001). BQ-123 restores the drug effect on CLL cells (*p<0.01 for idelalisib, **p<0.001 for PD98059). In addition, CLL cells (n = 8) were cultured (panel D) alone in complete medium or (panel E) in contact with HUVEC layer (HC). Fludarabine was added at 1 µM. Cells were also treated with 100 nM ET-1 and, as indicated, pretreated with 0.1 µM BQ-123 (20 min). Histograms represent mean±SEM of the percentage of live cells at 48 h of 8 CLL patients evaluated in 4 independent experiments. Control is defined as CLL cells cultured without any treatment alone in panel D and in co-culture in panel E. Flow cytometric contour plots of one representative case are shown in panel F. The gates in the plots exemplify viable CLL cells (Annexin V-/PI-, circled). Of note, inhibition of fludarabine-induced apoptosis is evident in presence of ET-1 both in CLL cultured alone and in CLL in co-culture. Again, treatment with BQ-123 improves CLL sensitivity to fludarabine-mediated apoptosis. (*p<0.05, **p<0.01, ***p<0.001).

Small molecules that target kinases downstream the BCR have shown marked anti-tumor effects in clinical trials [16], [17]. We evaluated if the inhibition of CLL viability mediated *in vitro* by molecules targeting effector proteins of BCR engagement may be counteracted by ET-1 signaling. To this end, we treated CLL cells with idelalisib at 0.5 µM or PD98059 at 50 µM with or without 100 nM ET-1 peptide. As shown in Figure 3, ET-1 signaling attenuated the pro-apoptotic effect of both idelalisib and PD98059. In particular, idelalisib decreased the CLL viability from 70±4% to 57±5% (n = 8, p = 0.002). The stimulation with ET-1 peptide reduced idelalisib effect (62±4%, p = 0.004), that was completely restored via ET_A_R inhibition (55±5%, p = 0.02) (Figure 3A). Likewise, the use of MEK inhibitor induced a significant decrease of cell viability in 8 CLL patients from 74±2% to 64±2% (p = 0.003). ET-1 signaling counteracted the effect of MEK inhibition, inducing an increase in CLL viability to 69±2% (p = 0.001). CLL cells were resensitized to MEK inhibition in the presence of BQ-123 (64±3%, p = 0.006, Figure 3B).

Lastly, we evaluated whether ET-1 could protect CLL cells from fludarabine-induced apoptosis. Leukemic cells from an independent subset of untreated CLL patients (n = 8) were also cultured for 48 hours alone or in contact with endothelial layer with the addition of fludarabine (1 µM) in presence or absence of ET-1 peptide at 100 nM. In blocking experiments, CLL cells were pre-incubated or not with BQ-123 for 20 minutes before treatments. We found a significant inhibition of drug-induced apoptosis in presence of ET-1 both in CLL cultured alone and in CLL in co-culture on endothelial layer, with an increase in viability from 25%±7% and 28%±6% in fludarabine-treated CLL to 31%±8% and 37%±6% in CLL treated with fludarabine and ET-1, respectively (p = 0.012 and p = 0.003, Figure 3). Again, treatment with BQ-123 was able to restore CLL sensitivity to fludarabine-mediated apoptosis (p<0.01, Figure 3). Blocking ET_A_R on CLL cells reversed the ET-1 mediated fludarabine-resistance both in mutated and unmutated IGHV subsets and also in 3 cases carrying unfavorable FISH aberrations (2 CLL with 11q deletion and 1 trisomy 12) (data not shown).

### ET-1 triggers CLL cell activation and proliferation

**Figure 4:**
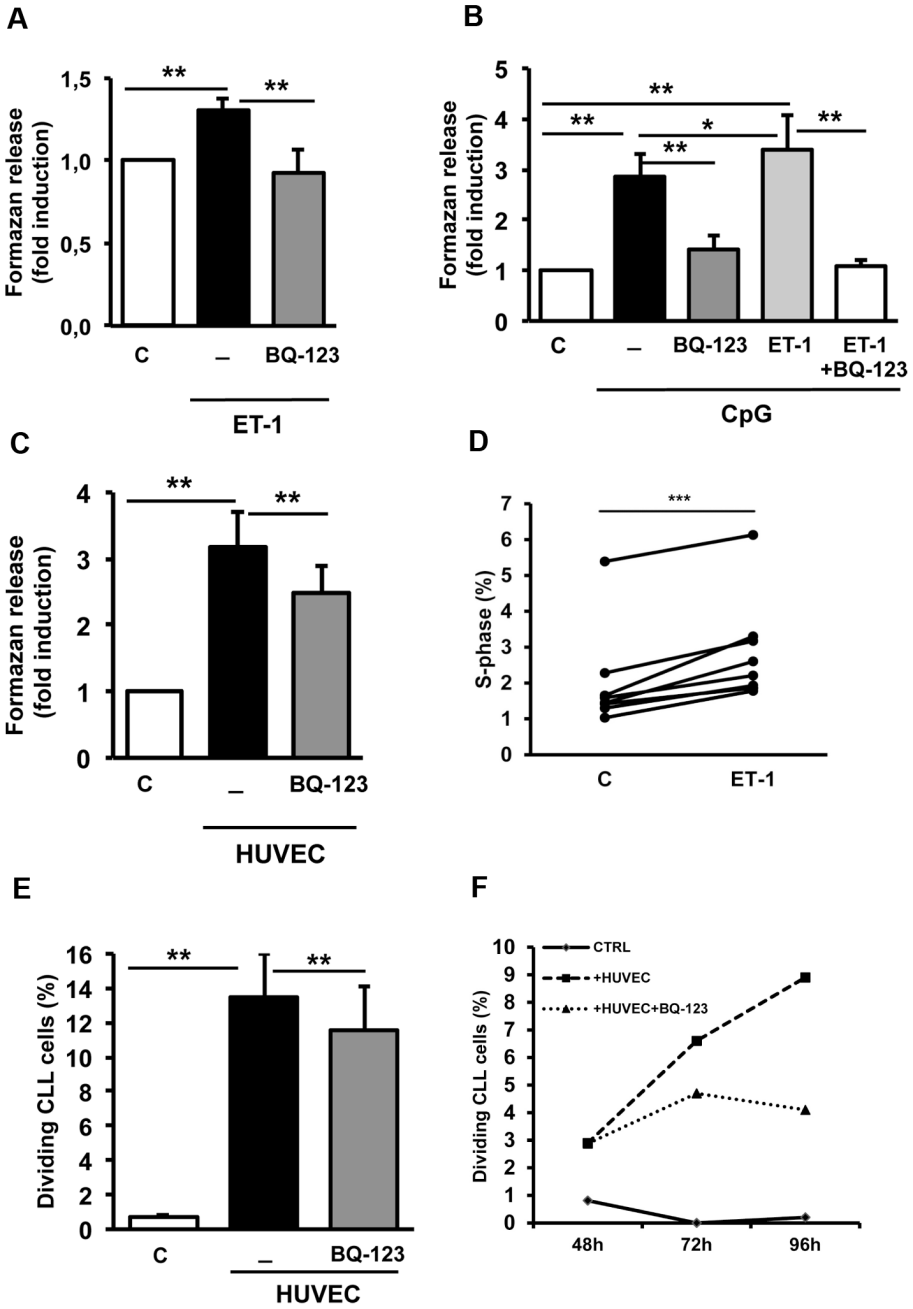
ET-1 induces CLL activation and proliferation through ET_A_ receptor. Purified CLL cells, pretreated or not with 0.1 µM BQ-123, were stimulated with 100 nM ET-1 for 4 hours (n = 7) in panel A, or with an agonist specific for toll-like receptor 9 (TLR9) (CpG oligonucleotides) and IL-2 with/without ET-1 addition for 5 days (n = 15) in panel B, or with endothelial cell contact (HC) for 4 days (n = 11) in panel C. Histograms depict the formazan release by metabolically active CLL cells as fold change compared to unstimulated control (untreated CLL cells cultured alone). Data are presented as mean±SEM of 6 independent experiments. Of note, the CLL activation is significantly increased upon ET-1 stimulation and inhibited by blocking ET_A_R signaling in all conditions. (D) After 1 h of serum-starvation, CLL cells (n = 8) were stimulated or not with ET-1 overnight. PI analysis was performed to define cell cycle phases. Histograms represent the percentage of CLL cells in S-phase after treatment compared to unstimulated control. (E) CFSE-labeled CLL cells were cultured for 4 days alone in complete medium (control) or on endothelial layers. Where indicated, CLL cells were incubated for 20 min with 0.1 µM BQ-123 before co-culture. The proliferative measure was inspected for 4 days, gating the CD19+ live CLL cells. The histograms represent cumulative data at 96 hours of 5 independent experiments by using 9 CLL patients. Data are shown as mean values ± SEM of the percentage of dividing CLL cells. In panel F, the percentages of divided CLL cells at 48 h, 72 h and 96 h measured by CFSE dilution in a representative CLL sample are shown. HUVEC cells stimulate CLL cells to divide. The addition of BQ-123 counteracts the EC-mediated proliferative stimuli. (*p<0.05, **p<0.01, ***p<0.001).

To explore the effect of ET-1 signaling pathway on CLL cell activation, we stimulated serum-starved CLL cells (n = 7) with 100 nM ET-1 for 4 hours, then measuring the extent of metabolically active cells able to release formazan in MTT assays. As shown in Figure 4A, ET-1 stimulation determined a 1.3-fold increase in CLL cell activation, that was neutralized by blocking ET_A_R on leukemic cells (p<0.01 both). Moreover, CLL cells (n = 15) were stimulated with ET-1 in combination with an agonist specific for toll-like receptor 9 (TLR9) (CpG oligonucleotides) for 5 days. As shown in Figure 4B, CpG oligonucleotides determined a 2.9-fold increase in metabolically active CLL cells when added alone and a 3.4-fold increase when used in combination with ET-1 (p<0.01 both, compared to cells cultured alone in medium). The CLL activation was significantly inhibited by blocking ET_A_R signaling. Furthermore, we established a direct contact between CLL cells (n = 11), pretreated or not with BQ-123 0.1 µM, and endothelial layer for 4 days and then we measured the activation status of CLL cells. Endothelial cell-contact induced CLL cell activation with a 3.2-fold increase as compared to cells cultured alone (p<0.01). Floating HUVEC cells, derived from the spontaneous cell detachment from the adherent monolayer, did not show any formazan production, thus excluding that contaminating endothelial cells may determine the high levels of formazan release in co-culture condition. We found that CLL activation due to endothelial cell contact was significantly reduced by pretreating CLL with BQ-123 (p<0.01) (Figure 4C).

We then investigated whether ET-1/ET_A_R signaling may trigger CLL cell proliferation. As shown in Figure 4D, when CLL cells (n = 8) were stimulated with 100 nM ET-1 overnight, we detected a moderate but significant increase in the percentage of CLL cells in S-phase as compared to the untreated control (p = 0.0004). Furthermore, we cultured CFSE-stained CLL cells (n = 9) in contact with endothelial layer for 4 days, then testing proliferation by CFSE dilution assays on CD19+ alive CLL cells. CLL cell stimulation with CpG oligonucleotides/IL-2 was used as positive control (25% divided CLL cells). Contact with HUVEC cells not only improved the percentage of viable cells but also induced the division of 13.5%±2.5% of CLL cells (p = 0.001). Blocking ET_A_R on leukemic cells by BQ-123, cell proliferation was significantly decreased to 11.6%±2.5% (p = 0.002, Figure 4E and 4F). Accordingly, the increased percentage of Ki67+ CLL cells (from 1.3 to 5%) in contact with endothelial cells was reduced to 4.2% in presence of BQ-123 (n = 3, data not shown). Collectively, these data demonstrate that activation of ET-1 pathway induces a proliferative profile in CLL cells.

### Big ET-1 plasma levels are predictor of short Time to First Treatment (TTFT) in CLL

**Figure 5:**
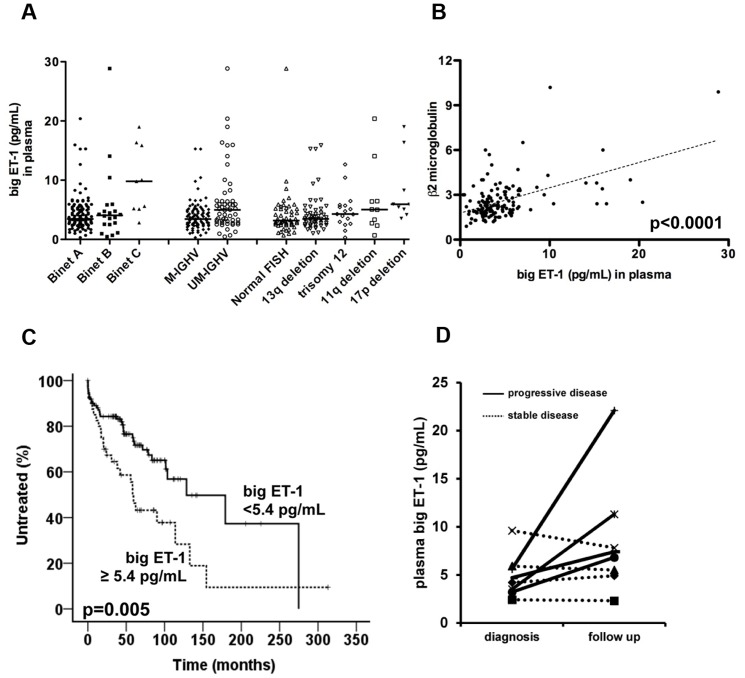
High levels of big ET-1 are associated with worse clinical outcome in CLL patients. (A) Dot plots depict the levels of big ET-1 in plasma samples collected at diagnosis from 151 CLL patients in relation to Binet stages, IGHV mutational status and FISH aberrations. Note that big ET-1 levels increase in patients with advanced stages, unmutated IGHV genes and unfavorable FISH aberrations (p<0.05 in all instances). (B) Positive correlation between big ET-1 levels and β2 microglobulin is represented (p<0.0001). (C) Kaplan-Meier curves for time to first treatment (TTFT). Patients are stratified in high and low big ET-1 subsets based on a cut-off equal to 5.4 pg/mL. CLL patients with high levels of big ET-1 display significantly shorter TTFT (p = 0.005; log-rank test). (D) Measurement of big ET-1 plasma levels in two plasma samples (diagnosis and follow up) collected from 8 CLL cases. Dotted lines represent CLL patients with stable disease during follow up, whereas dashed lines depict CLL patients showing progressive disease. Note that increase of big ET-1 plasma levels is measured in patients experiencing disease progression (n = 4, 4.3 pg/mL at diagnosis and 11.9 pg/mL pre-treatment).

We measured the levels of ET-1 precursor (big ET-1 peptide) in plasma samples collected at diagnosis from a multicentric cohort of CLL patients (n = 151). Patients' characteristics are summarized in Table S1 in File S1. Big ET-1 levels ranged from 0.3 pg/mL to 28.9 pg/mL (median = 3.7 pg/mL). As shown in Figure 5, higher levels of big ET-1 were detected in patients with advanced Binet stage (median, 3.5, 4.2 and 9.8 pg/mL in stage A, B and C respectively, p = 0.004), unmutated IGHV status (median, 3.4 and 5.0 pg/mL in mutated and unmutated IGHV CLL subsets, p = 0.003), intermediate/high FISH risk (median, 3.3 and 5.1 pg/mL in low and high FISH risk subsets, p = 0.002). In particular, a progressive increase in big ET-1 levels characterized CLL with hierarchically ranked FISH abnormalities (3.2, 3.5, 4.3, 5.0 and 5.9 pg/mL in CLL with normal FISH, 13q deletion, trisomy 12, 11q and 17p deletions respectively) (Figure 5A). No differences in big ET-1 levels were measured inside CD38 and ZAP-70 CLL subsets. Notch1 mutation (c.7544_7545delCT p.P2515fs*4) was detected in 9 patients, and TP53 mutations in 6 patients. Although not reaching statistical significance, we found that increased levels of big ET-1 were also present in patients harboring Notch1 and TP53 mutations compared to other cases (median, 4.3 vs. 3.7 pg/mL for Notch1; 5.5 pg/mL vs. 3.7 for TP53). A positive correlation was detected between big ET-1 levels and lymphocyte count (p<0.0001) or β2 microglobulin (p<0.0001) (Figure 5B).

Furthermore, we evaluated whether higher big ET-1 levels may characterize CLL patients with adverse clinical outcome. We found that patients with big ET-1 levels higher than a cutoff point of 5.4 pg/mL (established by ROC analysis) showed shorter time to first treatment (TTFT), as compared to CLL with low levels of big ET-1 (median TTFT, 58 vs. 129 months, p = 0.005, Figure 5C). Lastly, we performed a comparison of big ET-1 levels between two sequential PB plasma samples collected from 8 CLL cases with median interval of 5 years (range, 1–6 years). Four CLL patients showed stable disease during follow up, whereas the remaining cases were characterized by progressive disease. As shown in Figure 5D, no difference in big ET-1 levels was found in cases with stable disease during follow up. Conversely, increase in big ET-1 plasma levels over time was measured in patients experiencing disease progression (n = 4, 4.3 pg/mL at diagnosis and 11.9 pg/mL pre-treatment).

## Discussion

ET-1 was discovered as a potent vasoconstrictor, but later it was demonstrated to possess a wide range of pleiotropic functions, including cell survival, proliferation, angiogenesis, and regulation of tumor-infiltrating immune cells, invasion and metastasis [12], [18]. These actions are mediated through the ET_A_ receptor, whereas the triggering of ET_B_ receptor counteracts these functions in many cases [19], [20].

ET-1 is synthetized and secreted by human endothelial cells, many epithelial cell types, peripheral blood monocytes, differentiated macrophages, and mature dendritic cells [18], [20], [21]. ET-1 is also reported to be expressed by several tumor cell lines and primary solid neoplasia [22], [23]. In contrast, endothelin-1 is undetectable in unstimulated B and T lymphocytes or neutrophils and in several cell lines from hematological malignancies [21], [22], [24], [25]. We demonstrated for the first time that CLL cells circulating in peripheral blood and infiltrating lymph nodal compartments synthetize ET-1 peptide and express ET_A_ receptor on cellular surface. Leukemic cells expressed higher levels of ET-1 than normal B cells both in the peripheral blood and in lymph nodes. Again, CLL cells showed increased amount of ET_A_R as compared to circulating B cells from healthy donors. The difference seems particularly impressive at transcriptional levels and when total ET_A_R protein expression was detected by western blot, but to a lesser extent when measured on the cell surface. One possible explanation would be that ET-1 binding to ET_A_R on CLL cells promotes receptor internalization. Upon ET-1 binding, ET receptors have been shown to form homodimers and heterodimers, and to accumulate in the cell interior, then subsequently become sorted to distinct cellular fates. ET_A_ is typically recycled back to the plasma membrane in an un-liganded state, whereas ET_B_R is targeted to lysosomes for degradation [26]. Further studies will be necessary to elucidate these mechanisms that can have profound effects on ligand binding, receptor activation, desensitization, and membrane trafficking in CLL cells. ET-1 peptide, measured as the 38-aa precursor big ET-1, was detected in conditioned media collected from CLL cells, and also accumulated at high levels when leukemic cells were cultured in direct contact with endothelial cells. Conversely, normal B cells did not secrete ET-1 in vitro. Overall, our findings suggested that endothelin-1 signaling may be abnormally activated in leukemic cells compared to normal B cells.

ET-1 is a known survival factor for many normal and tumoral cell types, acting mainly through ET_A_ receptor [12], [18], [27]. We argued whether ET-1 signaling pathway may trigger survival stimuli on CLL cells by establishing an autocrine loop and/or by acting throughout microenvironment. We observed an enhanced resistance to spontaneous apoptosis in CLL cells cultured with recombinant ET-1. The effect was reversed by blocking ET_A_ receptor with the selective antagonist BQ-123, meaning that ET-1-mediated ET_A_R activation triggers protective and antiapoptotic signals on CLL cells. Accordingly, we demonstrated that ET-1 activates PI3 kinase and MAP kinase signaling pathways in CLL cells throughout ET_A_R triggering. Signals from the tumor microenvironment play a pivotal role in the maintenance and survival of CLL cells. In particular, BCR signaling has been recognized as an essential signal for CLL selection and expansion [28]–[31]. Engagement of BCR mediates Btk phosphorylation, which in turn activates several downstream signaling molecules such as PI3K and MEK protein kinases. Inhibitors of kinases involved in BCR signal transduction have demonstrated substantial clinical activity in CLL [16], [17]. The fact that ET-1 and BCR signals converge to common downstream pathways may be of interest. Here, we found that blocking ET_A_R via BQ-123 interferes with ERK phosphorylation and CLL pro-survival effect mediated by BCR activation.

We and others recently demonstrated that the contact with endothelial cells rescues CLL from spontaneous and drug-induced apoptosis, induces activation and proliferation and generates a peculiar gene expression profile on leukemic cells [6], [8], [10], [32]. ET-1 was observed among the most up-regulated genes in CLL after co-culture and is also secreted at high amount by activated endothelium. In addition, interaction with endothelial cells improved CLL survival by physical contact throughout β_1_- and β_2_- integrins but also by secretion of soluble factors [8]. As consequence, we argued whether ET-1 may be involved in CLL/endothelial cell crosstalk. We demonstrated that the blockade of ET_A_R on CLL cells significantly reduces apoptosis-resistance acquired by CLL cells after contact with endothelial layer.

We also evaluated whether ET-1 signaling may determine protection against drug-induced apoptosis as reported in solid tumors [33]–[35]. We found that ET-1 reduces the cytotoxic effect of fludarabine on CLL cells cultured alone in complete media or co-cultured on endothelial layers. ET_A_R blockade by BQ-123 antagonist inhibited the ET-1-mediated protection against fludarabine-induced apoptosis. Given the interconnected signaling network of ET-1, it is also important to explore the potential value of combinatorial therapies with signal transduction inhibitors such as lipid kinase PI3Kδ inhibitor idelalisib and MEK inhibitor PD98059. Here, we found that ET-1 signaling decreased the pro-apoptotic effect of both molecules. The combination with BQ-123 completely neutralized the protective effect of ET-1. Remarkably, despite the CLL heterogeneous sensitivity to MEK inhibition [36], the blockade of ET_A_R restored the CLL sensitivity to PD98059 in all CLL cases. In this scenario, the ET-1 peptide secreted by CLL cells or by other cell types, such as endothelial cells inside infiltrated tissues, could interfere with the effect of novel molecules currently undergoing clinical trial with promising results [16], [37].

It has been reported that ET-1 stimulates mitogenic responses and expression of proto-oncogenes in normal cell types (vascular smooth muscle cells, fibroblast, and glomerular mesangial cells) and also in several human cancer cell lines and primary tumor cells [38]–[41]. We demonstrated that ET_A_R triggering mediates proliferative stimuli on CLL cells, including activation of MAP kinase signaling pathway, cell cycle progression and increased number of divided cells. The blockade of ET_A_ receptor on CLL cells by BQ-123 reduced the extent of proliferating subclone that resulted by intimate contact with endothelial cells. These findings support the view that ET-1 could participate in the maintenance and progression of leukemic clone inside tissues. Although the majority of circulating CLL cells are quiescent, a small proliferative compartment does exist in CLL conceivably within the lymph nodes and bone marrow, where leukemic cells may take advantage of interactions with the microenvironment [1], [42]. Intimate contact with surrounding non-transformed cells, extracellular matrix elements and soluble factors affect CLL-cell survival and proliferation, induce genetic instability and contribute to clonal evolution [2], [30]. In this scenario, ET-1/ET_A_R axis may be a relevant player in maintaining CLL clone by inducing apoptosis resistance and protection against drug effects, and also by providing growth and proliferative stimuli inside microenvironmental tissues.

Elevated plasma levels of ET-1 were detected in patients diagnosed with various solid tumors and may be useful in predicting survival [43]–[48]. Due to low circulating concentration and short plasma half-life (∼1.5 minutes), measurement of ET-1 21-residue peptide in plasma has proven to be difficult. Big ET-1 is a stable peptide with a half-life of 30 minutes in plasma and may represent a sensitive and valuable indicator of endothelin system activation [49]. We measured the levels of big ET-1 in plasma samples collected at diagnosis from a multicentric cohort of 151 CLL patients. Increased levels of big ET-1 characterized patients with advanced clinical stages, unmutated IGHV genes, higher lymphocyte count and β2 microglobulin levels. Moreover, a progressive increase in big ET-1 levels was detected in CLL harboring high risk FISH abnormalities, i.e. 17p and 11q deletions. Noteworthy, patients with higher levels of big ET-1 in plasma showed shorter time to first treatment. In agreement, patients with stable disease did not experience any increase in big ET-1 overtime, whereas higher amount of big ET-1 compared to diagnosis could be measured in patients with disease progression. The results support the notion that the activation status of endothelin system may be of relevance in CLL clinical outcome.

Several issues concerning the role of endothelin system in CLL remain to be explored. First, multiple mechanisms both at transcriptional and post-translational levels may be implicated in the abnormal levels of ET-1 and ET_A_R on CLL cells as compared to normal B cells. Second, the mechanisms underlying ET-1-induced mitogenesis involve the activation of several pathways, including the production of second messengers, calcium release, and synergism with growth factors such as interleukin-6, basic fibroblast growth factor and vascular growth factor. Furthermore, there is experimental evidence from other cellular systems, mainly smooth muscle cells, that the ET-1/ET_A_R axis is functionally associated to CD38 and the localization in well-defined area of the cell membrane is critical for the activation of CD38 and ET-1/ET_A_R pathways [50]–[52]. Interestingly, CLL cells also expressed ET_B_R on the cell surface (data not shown), even if at lower levels as compared to ET_A_R. Generally, ET_B_ receptor activation operates in a counter-regulatory fashion to ET_A_R and leads to cell apoptosis, but in some cell types ET_B_R was reported to mediate cell survival. Here, we deeply investigated the pleiotropic actions of ET_A_ receptor triggering in CLL cells. Further studies are needed to clarify the role of ET_B_ receptor. In view of the promising activity of the dual ET_A_R and ET_B_R antagonists in preclinical models of ovarian cancer, and the well-tolerated toxicity profile [53], [54], these molecules might be explored in CLL in combination with chemotherapy and kinase inhibitors. In conclusion, our data show for the first time that CLL cells produce ET-1 and express ET receptors at higher levels compared to normal B lymphocytes. The results also demonstrate that ET-1/ET_A_R axis plays a role in survival, drug-resistance and proliferation of leukemic cells. The observed ability of ET_A_R selective antagonist to interfere with intrinsic and extrinsic growth/protective signals of CLL cells may be explored, both experimentally and clinically, as a possible novel therapeutic approach in CLL.

## Supporting Information

File S1 **Contains the files: Figure S1. ET-1 expression in mutated vs. unmutated IGHV CLL subsets.** (A) ET-1 expression levels were evaluated by quantitative reverse-transcription PCR on mutated IGHV CLL (n = 3) and unmutated IGHV CLL (n = 7) cells purified from peripheral blood. Histograms depict mean±SEM of ET-1 relative expression. Results were normalized to the expression of GAPDH housekeeping gene. No differential expression of ET-1 mRNA is evident between the two subsets. (B) Big ET-1, the 38-aa precursor of ET-1, was quantified by ELISA in conditioned media obtained after 72 h-culture from 4 mutated IGHV CLL and 9 unmutated ones. Histograms depict mean±SEM of big-ET-1 levels in pg/mL. Unmutated CLL cells secrete higher levels of big-ET-1 as compared to mutated CLL (*p<0.05). **Figure S2. ET-1 signaling improves CLL survival and promotes fludarabine resistance.** (A) CLL cells (n = 6), pre-treated or not with 0.1 µM or 1 µM BQ-123, were stimulated with 100 nM ET-1. Viability was inspected by flow cytometry using Annexin-PI staining. Histograms represent mean±SEM of the percentage of viable cells (Annexin V-/PI-) in 3 independent time course experiments from 48 h to 96 h. ET-1 stimulation improves CLL viability at 96 h when leukemic cells decrease their spontaneous apoptosis resistance in vitro. (B) CLL cells (n = 11), pre-treated or not with 0.1 µM or 1 µM BQ-123, were cultured in contact with endothelial layers. Viability was inspected by flow cytometry using Annexin-PI staining. Histograms represent mean±SEM of the percentage of viable cells (Annexin V-/PI-) in 4 independent time course experiments from 48 h to 96 h. The blockade of ET_A_R by BQ-123 affects EC-mediated survival advantage at 72 h and 96 h. CLL cells (n = 8) were cultured (panel C) alone in complete medium or (panel D) in contact with HUVEC layer (HC). Fludarabine was added at 1 µM. Cells were also treated with 100 nM ET-1 and, as indicated, pretreated with 0.1 µM BQ-123 (20 min). Histograms summarize data at 24 h and 48 h, showing ET-1 mediated fludarabine-resistance at 48 hours. Control is defined as viability of CLL cells cultured alone in complete medium in panels A, B and C or in co-culture in panel D. (*p<0.05). **Figure S3. The blockade of ET_A_R by BQ-123 induces apoptosis on both mutated IGHV and unmutated IGHV CLL subsets.** (A) CLL cells (n = 6, 3 mutated IGHV and 3 unmutated IGHV CLL), pre-treated or not with 0.1 µM or 1 µM BQ-123, were stimulated with 100 nM ET-1. (B) CLL cells (n = 11, 4 mutated IGHV and 7 unmutated IGHV CLL), pre-treated or not with 0.1 µM or 1 µM BQ-123, were cultured in contact with endothelial layers. Viability was inspected by flow cytometry using Annexin-PI staining. Histograms represent mean±SEM of the percentage of viable cells (Annexin V-/PI-) at 96 h of CLL divided into mutated vs. unmutated IGHV subsets. Control is defined as viability of CLL cells cultured alone in complete medium. (*p<0.05, **p<0.01). **Table S1.** Patients' characteristics (n = 151).
(DOCX)
