# Supplementary material for: Endothelin-1 Promotes Survival and Chemoresistance in Chronic Lymphocytic Leukemia B Cells through ETA Receptor
Source: PLoS One. 2014 Jun 5;9(6):e98818. doi: 10.1371/journal.pone.0098818 (PMC4046988; doi:10.1371/journal.pone.0098818)
Supplement: File S1 — Contains the files: Figure S1. ET-1 expression in mutated vs. unmutated IGHV CLL subsets. (A) ET-1 expression levels were evaluated by quantitative reverse-transcription PCR on mutated IGHV CLL (n = 3) and unmutated IGHV CLL (n = 7) cells purified from peripheral blood. Histograms depict mean±SEM of ET-1 relative expression. Results were normalized to the expression of GAPDH housekeeping gene. No differential expression of ET-1 mRNA is evident between the two subsets. (B) Big ET-1, the 38-aa precursor of ET-1, was quantified by ELISA in conditioned media obtained after 72 h-culture from 4 mutated IGHV CLL and 9 unmutated ones. Histograms depict mean±SEM of big-ET-1 levels in pg/mL. Unmutated CLL cells secrete higher levels of big-ET-1 as compared to mutated CLL (*p<0.05). Figure S2. ET-1 signaling improves CLL survival and promotes fludarabine resistance. (A) CLL cells (n = 6), pre-treated or not with 0.1 µM or 1 µM BQ-123, were stimulated with 100 nM ET-1. Viability was inspected by flow cytometry using Annexin-PI staining. Histograms represent mean±SEM of the percentage of viable cells (Annexin V-/PI-) in 3 independent time course experiments from 48 h to 96 h. ET-1 stimulation improves CLL viability at 96 h when leukemic cells decrease their spontaneous apoptosis resistance in vitro. (B) CLL cells (n = 11), pre-treated or not with 0.1 µM or 1 µM BQ-123, were cultured in contact with endothelial layers. Viability was inspected by flow cytometry using Annexin-PI staining. Histograms represent mean±SEM of the percentage of viable cells (Annexin V-/PI-) in 4 independent time course experiments from 48 h to 96 h. The blockade of ETAR by BQ-123 affects EC-mediated survival advantage at 72 h and 96 h. CLL cells (n = 8) were cultured (panel C) alone in complete medium or (panel D) in contact with HUVEC layer (HC). Fludarabine was added at 1 µM. Cells were also treated with 100 nM ET-1 and, as indicated, pretreated with 0.1 µM BQ-123 (20 min). Histograms summarize data at [file pone.0098818.s001.docx]

**A**

**B**

*****

**Figure S1**

**B**

**A**

*****

*****

*****

**D**

*****

*****

**C**

*****

**Figure S2**

*****

*****

*****

*****

******

**C**

**-**

**0.1µM**

**BQ-123**

**1µM**

**BQ-123**

**A**

**ET-1**

******

**B**

*****

*****

*****

******

*****

**C**

**-**

**0.1µM**

**BQ-123**

**1µM**

**BQ-123**

**HUVEC**

**Figure S3**

**Table S1.** Patients’ characteristics (n=151)

| Characteristic | No. of Patients (n = 151) | % |
| --- | --- | --- |
| Age, years |  |  |
| *Median* | 66 | |
| *Range* | 32-86 | |
| Sex |  |  |
| *Male* | 82 | 54% |
| *Female* | 69 | 46% |
| Binet stage (n=148) |  |  |
| *A* | 122 | 83% |
| *B/C* | 26 | 17% |
| IGHV mutational status (n=144) |  |  |
| *Mutated (≥2%)* | 90 | 63% |
| *Unmutated (<2%)* | 54 | 37% |
| CD38 (n=143) |  |  |
| *CD38 negative (<30%)* | 105 | 73% |
| *CD38 positive (≥30%)* | 38 | 27% |
| ZAP-70 (n=121) |  |  |
| *ZAP-70 negative (<20%)* | 61 | 50% |
| *ZAP-70 positive (≥20%)* | 60 | 50% |
| FISH abnormalities (n=142) |  | |
| Low risk | 105 | 74% |
| *Normal FISH* | 52 | 36% |
| *13q deletion* | 53 | 37% |
| Intermediate/high risk | 37 | 26% |
| *Trisomy 12* | 17 | 12% |
| *11q deletion* | 10 | 7% |
| *17p deletion* | 10 | 7% |
| β_2_ microglobulin (mg/L) (n=140) |  |  |
| *Low level (<2.2)* | 65 | 46% |
| *High level (≥2.2)* | 75 | 54% |
| First treatment (n=151) |  | |
| *No* | 92 | 61% |
| *Yes* | 59 | 39% |
| Survival (n=151) |  |  |
| *Alive* | 139 | 92% |
| *Dead* | 12 | 8% |

*Abbreviations: IGHV, immunoglobulin variable heavy gene;*

*ZAP-70, zeta-chain-associated protein kinase 70;*

*FISH, fluorescence in situ hybridization;*
